# Une ostéo-arthropathie hypertrophiante (OAH) secondaire ou syndrome de Pierre-Marie Bamberger révélant un adénocarcinome bronchique

**DOI:** 10.11604/pamj.2017.27.118.9084

**Published:** 2017-06-15

**Authors:** Fatima Zahra El M’rabet, Hajar Ouahbi

**Affiliations:** 1 Service d’Oncologie Médicale, CHU Hassan II, Fès, Maroc

**Keywords:** Adénocarcinome pulmonaire, ostéo-arthropathie hypertrophiante, syndrome, Pulmonary adenocarcinoma, hypertrophic osteoarthropathy, syndrome

## Image en médecine

Nous rapportons le cas d’une patiente jeune âgée de 33 ans qui présente depuis 2 ans des arthralgies avec des œdèmes des quatre membres aggravés par la survenue d’un hippocratisme digital et une ostéo-arthropathie hypertrophiante (OAH) secondaire ou syndrome de Pierre-Marie Bamberger. Dans le cadre du bilan étiologique, un scanner thoraco abdominal a objectivé la présence d’une volumineuse masse tissulaire d'allure tumorale de 11.5 cm massivement excavée pulmonaire au niveau du lobe supérieur droit avec large extension locorégionale et à distance. L’aspect histologique et immuno histochimique a été compatible avec un adénocarcinome pulmonaire primitif. La patiente a été mise sous chimiothérapie palliative avec début de régression des œdèmes. L’ostéo-arthropathie hypertrophiante ou syndrome de Pierre-Marie Bamberger est un syndrome paranéoplasique qui peut précéder un carcinome brochique dans 25% des cas. Il associe un hippocratisme digital (déformation des doigts en baguette de tambour, ramollissement de la matrice unguéale, et l’ongle, avec bombement et une déformation en verre de montre) et une augmentation de volume des articulations des os des mains et des pieds. Sur le plan radiologique, il se manifeste par un développement accru du périoste au niveau des diaphyses distales des os longs et au niveau des phalanges. Ces déformations ostéo-articulaires sont douloureuses, associées à des désordres neuro-vasculaires avec œdème important pouvant être responsable d’une impotence fonctionnelle. Le traitement étiologique suffit à faire régresser les déformations et les œdèmes comme le cas de cette patiente.

**Figure 1:**
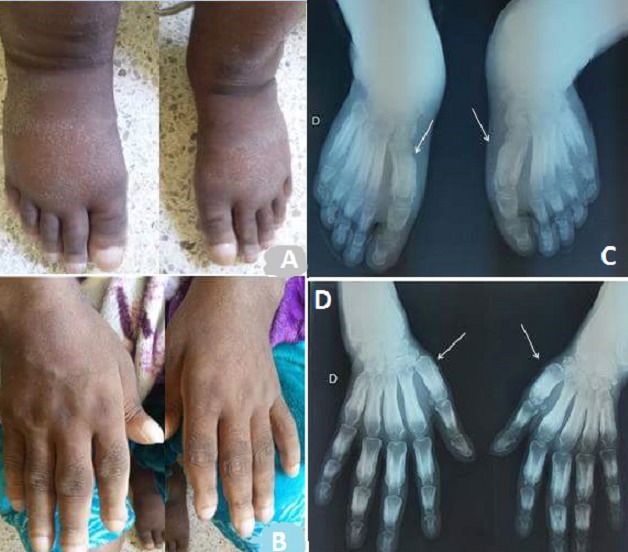
(A, B) un syndrome acromélique des mains et des pieds: comportant un hippocratisme digital, une hypertrophie des parties molles et des troubles vasomoteurs (œdème important avec cyanose); (C, D) les radiographies standards des mains et des avant pieds: une apposition périostée lamellaire des phalanges proximales avec infiltration importante des parties molles

